# Comparative Genomic Analysis of Coding Sequence-Derived Microsatellites Reveals Evolutionary Conservation and Genetic Diversity in Forest Musk Deer (*Moschus berezovskii*) and Related Ruminants

**DOI:** 10.3390/vetsci13080808

**Published:** 2026-08-15

**Authors:** Zhi-Jiang Dong, Ying-Ying Ren, Wen-Hua Qi

**Affiliations:** 1 College of Biology and Food Engineering, Chongqing Sanxia University of Science and Technology, Chongqing 404100, China; 2 College of Environmental and Chemical Engineering, Chongqing Sanxia University of Science and Technology, Chongqing 404100, China; 3 Chongqing Three Gorges Academy of Agricultural Sciences, Chongqing Sanxia University of Science and Technology, Chongqing 404020, China

**Keywords:** *Moschus berezovskii*, microsatellites, coding sequences, comparative genomics, KEGG enrichment, genetic diversity

## Abstract

The FMD is an endangered species under first-class national protection in China. Comparative genomic investigation of microsatellite (SSR) in CDS may provide insights into adaptive evolutionary mechanisms and may inform conservation management strategies for captive populations. Here, we analyzed the FMD genome alongside five closely related ruminants: cattle (*Bos taurus*), red deer (*Cervus elaphus*), white-tailed deer (*Odocoileus virginianus*), sheep (*Ovis aries*), and goat (*Capra hircus*). Through genome-wide bioinformatic identification, we systematically compared the abundance, density, structural categories, repeat motifs, chromosomal distribution, and pathway enrichment analysis of SSR-containing genes in CDS. Furthermore, we performed synteny analysis and evaluated population genetic diversity. A total of 2509 SSRs in CDS were identified in the FMD, with a relative density of 62.61 loci/Mb. Trinucleotide SSRs were overwhelmingly dominant (88.46%) in the FMD. Notably, the FMD exhibited the highest relative abundances of both tetranucleotide and pentanucleotide repeats among the six species (2.37 and 2.18 loci/Mb, respectively), with pentanucleotide abundance approximately 5.6- to 9.1-fold higher than that of the other species. Chromosomal mapping revealed the highest SSR density in CDS regions on chromosome 27, while SSR-containing genes exhibited a heterogeneous pattern characterized by localized clustering. Synteny analysis demonstrated relatively conserved syntenic relationships between the FMD and goat, sheep, and cattle, with moderate conservation also observed with red deer and white-tailed deer, suggesting that SSR-containing genes in ruminants may remain highly conserved during chromosomal rearrangements. GO and KEGG analyses indicated that SSR-containing genes across all species were predominantly enriched in transcriptional regulation, RNA processing, and signal transduction pathways. Specifically, the FMD showed enrichment patterns associated with hypoxia response, mRNA processing, and epigenetic regulation, which may reflect lineage-specific transcriptional patterns, though the functional involvement of these SSRs remains to be experimentally validated. In addition, the five primer pairs screened in this study exhibited high polymorphism, with a mean polymorphism information content (PIC) of 0.93. The observed heterozygosity (Ho) was significantly lower than the expected heterozygosity (He), and the mean inbreeding coefficient (FIS) was 0.57, indicating heterozygote deficiency and an elevated risk of inbreeding in this captive FMD population. Collectively, our findings provide preliminary insights into the conserved patterns of microsatellite evolution and lineage-specific divergence in ruminants, offering a reference framework for comparative genomics and adaptive evolution research, as well as practical molecular markers for genetic management of captive populations.

## Simple Summary

The forest musk deer (FMD, *Moschus berezovskii*) is an endangered species native to China and is currently threatened by habitat loss and reduced genetic diversity. Microsatellites (SSRs) located in coding sequences can influence gene function and provide valuable information for evolutionary and conservation studies. In this study, we compared coding sequence-derived SSRs in the FMD with those of five related ruminant species, including red deer, white-tailed deer, cattle, sheep, and goat. We found that trinucleotide repeats were the dominant SSR type across all species, indicating strong evolutionary conservation. However, the FMD showed unique features, including an unusually high abundance of CCG motifs and distinct functional enrichment associated with hypoxia response, RNA processing, and epigenetic regulation. In addition, five highly polymorphic SSR markers were developed and applied to a captive population. Genetic analysis revealed substantial genetic variation but also clear signs of heterozygote deficiency and potential inbreeding. These findings improve our understanding of microsatellite evolution in ruminants and provide useful molecular tools for the conservation and genetic management of FMD populations.

## 1. Introduction

Microsatellites, also known as simple sequence repeats (SSRs) or short tandem repeats (STRs), are ubiquitous DNA sequences composed of tandemly repeated 1–6 bp motifs distributed throughout eukaryotic genomes [1,2,3]. SSRs are characterized by high polymorphism, codominant inheritance, and abundant genomic distribution, and they serve as robust tools for molecular marker development, genetic diversity assessment, and phylogenetic reconstruction [4,5]. Beyond their conventional utility as genetic markers, SSRs are increasingly recognized at the genomic level for their active involvement in modulating gene expression, maintaining chromosomal architecture, and driving adaptive evolution [6,7]. Accumulating evidence indicates that SSRs play critical roles within gene regulatory networks by influencing transcription factor binding, altering chromatin conformation, and regulating RNA processing [8]. Furthermore, interspecific divergence in SSR distribution profiles offers profound insights into evolutionary histories and ecological adaptation strategies [9].

Historically, genetic investigations have predominantly focused on non-coding SSRs. However, SSRs located within coding sequences (CDSs) have garnered substantial interest in recent years due to their potential to directly modulate protein structure and function [10,11]. Compared to their non-coding counterparts, these coding-region SSRs are subject to more stringent selective constraints [12]. Variations in repeat length can induce frameshift mutations, thereby disrupting protein translation and functional stability [13]. Consequently, over long evolutionary timescales, SSRs in CDSs typically exhibit heightened structural conservation and specific compositional biases [14]. In vertebrates, trinucleotide repeats are overwhelmingly enriched within coding regions [3]. Because their motif lengths are multiples of three, insertions or deletions (indels) of these repeat units preserve the open reading frame, exerting relatively minor effects on protein function [15,16]. Conversely, non-triplet SSRs are prone to inducing frameshifts that severely disrupt gene function, and are thus heavily suppressed by natural selection [17]. This distinct distribution pattern is a direct manifestation of purifying selection acting to maintain functional genomic stability [18].

Elucidating CDS-SSR patterns in mammalian groups of economic and conservation significance is therefore essential for understanding their adaptive evolution and genetic management. As an important mammalian group, ruminants occupy vital ecological and economic positions. Advances in genome sequencing have continuously improved genomic resources for cattle (*Bos taurus*), sheep (*Ovis aries*), and red deer (*Cervus elaphus*) [19,20,21], yet systematic comparative analyses of CDS-SSRs in ruminants remain scarce [22]. Among ruminants, the FMD represents a species of exceptional economic and conservation value endemic to China; the musk secreted by adult males is highly prized in traditional medicine [23,24]. Unfortunately, owing to long-term overhunting and habitat destruction, wild FMD populations have declined sharply, and the species is listed as a first-class nationally protected species in China. Captive breeding has consequently become a key conservation strategy [25]. However, captive populations commonly suffer from reduced genetic diversity and an elevated risk of inbreeding [26,27]. Limited founder numbers coupled with prolonged closed breeding reduce effective population size (N_e_) [28,29] and accentuate genetic drift, thereby eroding genetic variation. Such genetic erosion compromises long-term population viability and evolutionary adaptive potential. It also adversely affects individual growth performance, stress tolerance, and reproductive behavior [2,30,31]. These traits, together with other economically important characteristics such as behavioral phenotypes and secretion-related traits, are polygenic and depend on abundant genetic variation for their proper expression. From a phylogenetic perspective, the forest musk deer belongs to the family Moschidae within the order Artiodactyla, which represents a lineage closely allied to the suborder Ruminantia. The five comparator species selected in this study—cattle, sheep, and goat (family Bovidae), as well as red deer and white-tailed deer (family Cervidae)—collectively span the two major families of ruminants. This assemblage provides a robust comparative framework because these species share a common ancestor within the Pecora infraorder, yet exhibit varying degrees of evolutionary divergence from the FMD: the cervids (red deer and white-tailed deer) are considered more closely related to moschids based on molecular phylogenetic studies, whereas the bovids (cattle, sheep, and goat) represent a more distantly related but well-characterized lineage. The availability of high-quality reference genomes for all five species further ensures methodological comparability and facilitates the identification of conserved versus lineage-specific patterns in CDS-SSR evolution.

More importantly, because CDS-derived SSRs are located directly within functional gene regions, they may reflect not only neutral genetic variation among populations [32], but also represent candidate loci positioned in genomic contexts associated with gene expression regulation, protein structural modification, and adaptive responses to environmental pressures [7]. It should be emphasized that these positional associations are correlational and do not establish a causal role of SSRs in gene regulation; experimental validation (e.g., allele-specific expression assays or editing of repeat tracts) is required to test any functional effects. Consequently, a systematic survey of CDS-SSRs in the FMD may identify candidate genetic variants in regions implicated in physiological adaptation, behavioral regulation, and reproductive performance, providing a reference framework for future hypothesis-driven studies and molecular marker-assisted captive management strategies.

Based on this, the present study focused on the FMD as the primary research subject, with five closely related ruminants, cattle, red deer, white-tailed deer (*Odocoileus virginianus*), sheep, and goat (*Capra hircus*), selected as comparators, to systematically analyze the distribution characteristics of SSRs at the CDS. The study comprised the following aspects: (1) genome-wide identification of SSRs in the CDSs of the six species by using bioinformatic approaches, with analysis of their abundance, density, and structural categories [33]; (2) comparison of the repeat types and motif compositions of SSRs among species, to reveal their conservation and species specificity; (3) characterization of SSR distribution patterns and their differences at the chromosomal level; (4) investigation of the evolutionary relationships of SSR-containing genes across genomes through synteny analysis [34]; (5) elucidation of the potential functions of SSR-associated genes via GO and KEGG enrichment analyses [35]; and (6) screening of highly polymorphic SSR loci and genetic diversity analysis to assess the genetic status of the captive FMD population [36].

## 2. Materials and Methods

### 2.1. Data Acquisition and Sequence Preparation

The reference genome assembly and corresponding annotation files for the FMD were retrieved from the muskDB database (http://muskdb.cn/home/), while those for cattle, sheep, goat, red deer, and white-tailed deer were acquired from the NCBI database (https://www.ncbi.nlm.nih.gov/).

To ensure interspecific comparability and minimize bias from variable transcript numbers and annotation redundancy, all annotation files were pre-processed prior to CDS extraction. Specifically, the “Representative GXF Obtain” function implemented in TBtools v2.530 was used to filter each GFF file, retaining only the longest protein-coding transcript isoform per gene locus as the representative transcript. This step effectively removes redundant alternative splice variants and fragmented gene models, yielding a standardized, non-redundant gene set for each species. Consequently, all downstream comparative analyses were based on comparable annotation sets in which each protein-coding gene was represented by a single, consistent transcript.

### 2.2. Identification and Classification of SSRs in CDS

Genome-wide identification of mono- to hexanucleotide SSRs within the CDSs of the FMD and the five comparator ruminants was performed primarily using MSDB v2.4 (Microsatellite Search and Building Database). The search parameters were configured as follows: (i) search mode—Perfect Search (for P-SSRs), supplemented by the Imperfect and Compound modules (for IP-, ICD-, ICX-, CD-, and CX-SSRs); (ii) motif scope—all motifs from mono- through hexanucleotides; (iii) minimum repeat thresholds—12, 7, 5, 4, 4, and 4 for mono-, di-, tri-, tetra-, penta-, and hexanucleotides, respectively; (iv) flanking sequence length—200 bp; (v) motif standardization—reverse-complementary and circularly permuted motifs were treated as equivalent (e.g., the trinucleotide motif AGC was standardized to represent its circular permutations GCA and CAG, as well as their reverse complements CTG, TGC, and GCT), with only the lexicographically smallest form retained to avoid redundancy; (vi) overlap handling—in cases of overlapping SSRs, the longest continuous repeat tract was retained; (vii) interrupted SSR criteria—IP-SSRs were defined as perfect repeat tracts interrupted by ≤10 bp of non-repeat sequence or ≤1 mismatch/indel; (viii) compound SSR distance—CD-SSRs and ICD-SSRs were defined when two or more distinct SSR motifs occurred within ≤10 bp of each other. Statistics were computed on a whole-sequence basis, with motif type, length, and repeat statistics displayed.

Krait was employed secondarily as an independent validation tool using parameters equivalent to those described above. Loci detected by both programs were retained; discrepancies were resolved through manual inspection of the raw sequence, with the MSDB output serving as the primary reference for final curation. SSRs were subsequently categorized as Perfect (P-SSRs), Interrupted Perfect (IP-SSRs), Interrupted Complex (ICX-SSRs), Interrupted Compound (ICD-SSRs), Complex (CX-SSRs), and Compound (CD-SSRs). Given the triplet nature of the genetic code, SSRs with repeat unit lengths divisible by three (i.e., tri- and hexanucleotides) were defined as triplet SSRs, whereas the remaining types (mono-, di-, tetra-, and pentanucleotides) were classified as non-triplet SSRs. To facilitate subsequent investigation of chromosomal distribution patterns, chromosome-level sequences were extracted for all studied species and used for SSR locus mapping.

### 2.3. Gene Ontology (GO) Functional Enrichment Analysis

To explore the potential biological functions of genes harboring SSRs within their CDS, the sequences of all SSR-containing genes identified in the FMD and the five comparative ruminant species were extracted and compiled in FASTA format. Gene IDs were then retrieved using TBtools. Functional enrichment analysis was performed using the DAVID database (https://davidbioinformatics.nih.gov/). GO terms were systematically classified into three principal categories: Molecular Function (MF), Cellular Component (CC), and Biological Process (BP). The resulting data were statistically analyzed and visualized using TBtools.

### 2.4. KEGG Pathway Enrichment Analysis

Functional annotation of SSR-containing genes within the CDS of FDM and the five comparative ruminants was performed using KAAS (KEGG Automatic Annotation Server, https://www.genome.jp/kegg/kaas/, accessed on 9 October 2025), with the mammalian gene set in the KEGG database serving as the reference background. Pathway enrichment analysis was subsequently conducted using TBtools. To ensure statistical robustness, *p*-values were adjusted for multiple testing using the Bonferroni correction, with an adjusted *p*-value < 0.05 set as the threshold for significant enrichment. This analysis aimed to elucidate the distribution of SSR-containing genes across major metabolic and signal transduction pathways, thereby providing insights into their potential biological functions.

### 2.5. Primer Design and Validation

Specific PCR primers were designed using Primer Premier 5.0 to target the previously identified trinucleotide SSRs within CDS regions. The expected amplicon sizes were strictly controlled between 200 and 400 bp, and critical parameters—including annealing temperature (Tm), GC content, and the potential for secondary structures (e.g., hairpins)—were comprehensively evaluated. Following commercial synthesis, the primers were experimentally validated via PCR amplification to identify those suitable for downstream population genetic analysis (Table 1).

### 2.6. Population Genetic Analysis

Following preliminary PCR screening, the amplified products were submitted to a professional genotyping service provider for fragment analysis and genotyping by capillary electrophoresis. The raw genotyping data were subsequently analyzed using specialized bioinformatics software to assess population genetic diversity. A rigorous quality control procedure was implemented to exclude loci with low amplification efficiency, low polymorphism, or null alleles, thereby ensuring that the ultimately selected SSR markers exhibited high stability and polymorphism. These markers thus provide robust molecular tools for the comprehensive genetic assessment of FMD populations.

## 3. Results

### 3.1. Genomic Characteristics of Coding Sequences in FMD and Related Ruminants

Genome-wide annotation of the FMD CDS identified a total of 24,375 genes, of which 1766 contained SSRs, yielding 2325 SSR loci in total. This gene count represents the largest coding repertoire among the six ruminant species examined, contrasting with the smaller CDS gene sets of white-tailed deer (20,293), goat (20,728), sheep (21,550), cattle (21,840), and red deer (23,212). In terms of SSR abundance, the FMD ranked third behind sheep (2547) and red deer (2477), but ahead of cattle (2295), white-tailed deer (2248), and goat (2176). Notably, despite an intermediate SSR count and a relative abundance (62.61 loci/Mb) that was surpassed by sheep (70.64), red deer (66.17), white-tailed deer (65.49), and cattle (63.32)—though comparable to goat (62.44)—the FMD exhibited the highest relative SSR density (1289.93 bp/Mb) among all species, alongside a CDS SSR content (0.13%) equivalent to sheep. This disparity between locus frequency and cumulative sequence density suggests that SSRs in the FMD coding regions are characterized by longer repeat tracts or more complex architectures, representing a species-specific genomic signature.

However, the FMD exhibited a remarkable capacity to harbor SSRs within individual genes. We identified up to 47 SSR loci within a single gene in this species, a value markedly higher than those observed in the comparative ruminants. Specifically, the maximum number of SSRs per gene was 15 in white-tailed deer, 13 in cattle, 12 in goat, and 9 in red deer; even in sheep, which displayed the highest count among the reference species, this number reached only 23. The occurrence of such extreme values strongly suggests a unique pattern of high SSR aggregation within certain functional genes in the FMD—a degree of clustering rarely observed in related ruminant species (Table 2).

### 3.2. Analysis of SSR Structural Types

In terms of structural classification, Perfect SSRs (P-SSRs) predominated in the coding sequences (CDSs) of the FMD and the other five ruminant species. In the FMD, P-SSRs accounted for 2325 loci, followed by other variant types in descending order: Interrupted Compound SSRs (ICD-SSRs, *n* = 37) > Interrupted Perfect SSRs (IP-SSRs, *n* = 30) > Compound SSRs (CD-SSRs, *n* = 14) > Interrupted Complex SSRs (ICX-SSRs, *n* = 9). No Complex SSRs (CX-SSRs) were detected in the FMD, a pattern shared with cattle and goat. In contrast, although extremely rare, CX-SSRs were detected in white-tailed deer (*n* = 1), red deer (*n* = 2), and sheep (*n* = 4), indicating that CX-SSRs, which represent the most structurally complex class, are generally rare in ruminant CDSs.

To eliminate the confounding effects of variations in genome size and total gene number, we calculated and compared the relative density (loci/Mb) of SSRs in CDSs across the six ruminant species. P-SSRs constituted the overwhelming majority of CDS-SSRs in all species, accounting for 96.3% of the total SSR density in the FMD. However, the P-SSR density in the FMD (62.61 loci/Mb) was not the highest among the six species; it ranked fifth, below sheep (70.64), red deer (66.17), white-tailed deer (65.49), and cattle (63.32), and was comparable to goat (62.44). Consequently, the overall SSR density in the FMD (65.04 loci/Mb) was intermediate, surpassing only goat (64.50) but falling below cattle (65.39), white-tailed deer (67.71), red deer (67.96), and sheep (73.16). Nevertheless, the FMD exhibited distinctive features in complex SSR categories. The densities of Interrupted Compound SSRs (ICD-SSRs, 1.00 loci/Mb) and Interrupted Complex SSRs (ICX-SSRs, 0.24 loci/Mb) were the highest among the six species. In contrast, the abundance of CD-SSRs in the FMD (0.38) was lower than that in sheep (0.44) but higher than that in goat (0.34). Notably, sheep exhibited the highest abundance of Interrupted Perfect SSRs (IP-SSRs, 0.97), compared to 0.88 in cattle, 0.81 in the FMD, and 0.86 in goat. Regarding the most complex CX-SSRs, the abundance was zero in the FMD, cattle, and goat, further confirming the absence of such highly complex repeat structures in the coding regions of these three species (Table 3).

In summary, rather than exhibiting the highest overall SSR density, the FMD is characterized by a unique landscape marked by exceptionally high densities of ICD-SSRs and ICX-SSRs alongside a moderate P-SSR density. Consequently, this study focuses on P-SSRs for subsequent analyses.

**Figure 1:**
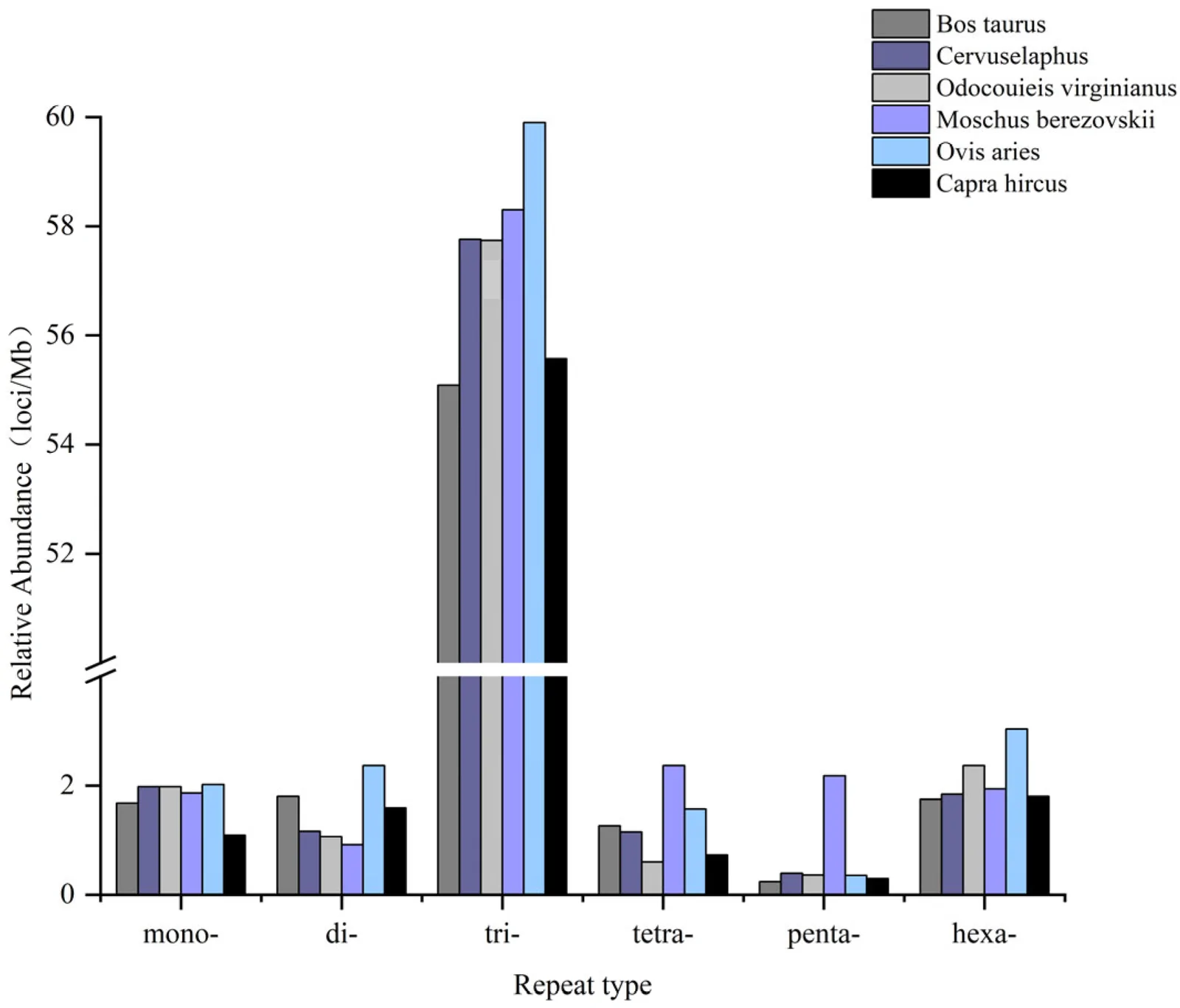
Comparison of SSR frequency of different repeat types in the CDS regions of the *M. berezovskii* and its closely related species.

Across the six ruminant species, Perfect SSRs (P-SSRs) within CDSs were overwhelmingly dominated by trinucleotide repeats, which constituted the largest class in all species, ranging from 55.09 loci/Mb in cattle to 59.90 loci/Mb in sheep; the FMD exhibited an intermediate-to-high level of 58.30 loci/Mb (Figure 1). When trinucleotide motifs were excluded, the remaining repeat classes exhibited marked interspecific variation. Notably, the FMD displayed a distinctive non-triplet profile characterized by the highest relative abundances of both tetranucleotide (2.37 loci/Mb) and pentanucleotide (2.18 loci/Mb) SSRs among the six species. These values substantially exceeded those observed in cattle (1.26 and 0.24 loci/Mb), white-tailed deer (0.60 and 0.36 loci/Mb), red deer (1.15 and 0.39 loci/Mb), sheep (1.57 and 0.35 loci/Mb), and goat (0.73 and 0.30 loci/Mb). In particular, the FMD pentanucleotide abundance was approximately 5.6- to 9.1-fold higher than that of the other species, representing the most pronounced species-specific deviation in the entire repeat-type spectrum.

By contrast, the FMD exhibited the lowest dinucleotide SSR abundance (0.92 loci/Mb) among the six species, falling below cattle (1.80), red deer (1.16), white-tailed deer (1.06), sheep (2.37), and goat (1.59). The other non-triplet classes showed variable rankings across the comparator species: hexanucleotide SSRs predominated among non-triplet repeats in white-tailed deer (2.37 loci/Mb), sheep (3.04 loci/Mb), goat (1.80 loci/Mb), and red deer (1.84 loci/Mb), whereas mononucleotide repeats were relatively prominent in red deer (1.98 loci/Mb) and sheep (2.02 loci/Mb). Red deer exhibited a distinct pattern in which mononucleotide abundance surpassed hexanucleotide, a ranking not observed in the other species.

### 3.3. Chromosomal-Scale Microsatellite Analysis

At the chromosomal scale, CDS-SSR densities were calculated for all assembled autosomes and the X chromosome as the number of CDS-SSRs divided by the total CDS length (bp) of each respective chromosome, multiplied by 10^6^. The Y chromosome was systematically excluded from interspecific comparisons because complete Y-chromosome assemblies are unavailable for two of the six species (Odocoileus virginianus and Cervus elaphus); this exclusion was applied uniformly across all species to ensure methodological consistency.

**Figure 2:**
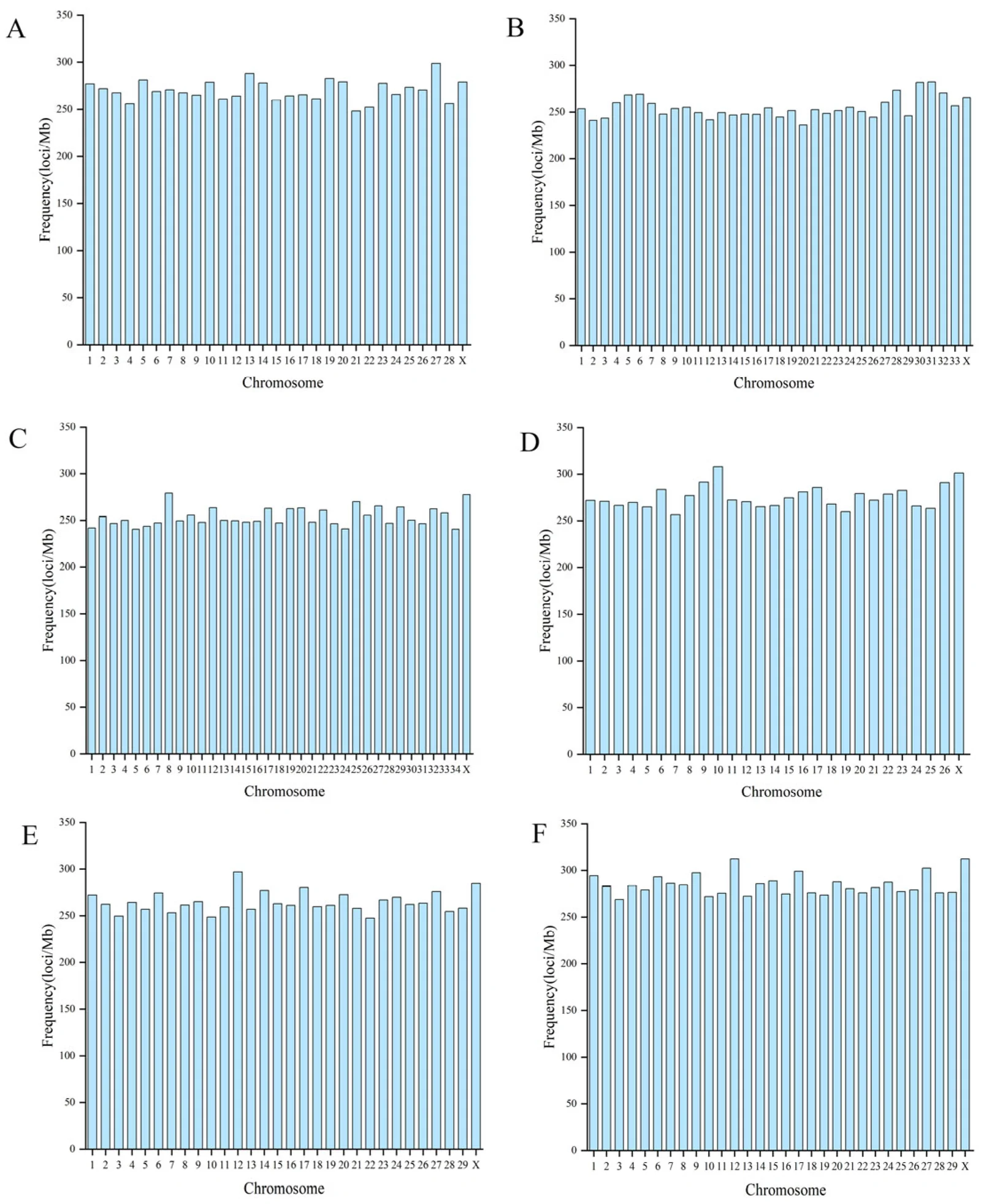
Comparison of chromosomal SSR abundance between the *M. berezovskii* and its related species, (**A**): *M. berezovskii*; (**B**): *Ce. elaphus*; (**C**): *Od. virginianus*; (**D**): *B. taurus*; (**E**): *Ov. aries*; (**F**): *Ca. hircus.*

Overall, CDS-SSR densities exhibited a relatively stable and comparable distribution pattern across the six ruminant species (Figure 2). In the FMD, chromosomal densities ranged from 248.26 loci/Mb (chromosome 21) to 298.77 loci/Mb (chromosome 27), reflecting moderate intra-genomic heterogeneity. This range was comparable to that observed in goat (247.35–296.8 loci/Mb) and sheep (256.7–307.91 loci/Mb), suggesting that bovid and cervid lineages share a broadly conserved pattern of chromosomal SSR distribution.

Among the comparator species, cattle displayed the highest peak density among all assembled chromosomes, reaching 312.32 loci/Mb on chromosome 12—surpassing the maximum observed in the FMD (298.77 loci/Mb). Sheep also exhibited a pronounced peak on chromosome 10 (307.91 loci/Mb), marginally exceeding the FMD maximum. By contrast, the two cervid species—white-tailed deer and red deer—showed relatively attenuated chromosomal CDS-SSR densities overall. White-tailed deer densities ranged from 240.38 loci/Mb (chromosome 5) to 279.24 loci/Mb (chromosome 8), while red deer ranged from 261.2 loci/Mb (chromosome 20) to 282.18 loci/Mb (chromosome 31), both falling below the peak values observed in the FMD, cattle, and sheep. Goat displayed the most even distribution across its 30 chromosomes, with the narrowest range (247.35–296.8 loci/Mb) and the lowest minimum density among the six species.

Collectively, these patterns indicate that CDS-SSR chromosomal distribution is characterized by broad evolutionary conservation across ruminants, with no single species exhibiting systematically elevated densities throughout the genome. Instead, peak densities appear to be chromosome-specific rather than species-specific, likely reflecting localized sequence composition or regional variation in CDS architecture rather than lineage-specific evolutionary trends.

### 3.4. Comparison of Repeat Copy Number Classes of Coding-Region SSRs Among the FMD and Related Ruminants

Across the six ruminant species, the motif abundance profiles of perfect SSRs within CDSs (P-SSRs) revealed that the FMD was broadly consistent with the other five species (cattle, goat, sheep, white-tailed deer, and red deer) in terms of the predominance of trinucleotide microsatellites. This suggests that the SSR composition within ruminant coding regions is highly conserved. Specifically, all six species exhibited a distribution pattern in which trinucleotide repeats were overwhelmingly dominant, while mono-, di-, and tetranucleotide repeats occurred at comparatively lower frequencies. Among the trinucleotide SSRs, CCG, AGG, and AGC consistently constituted the predominant core motifs, while ACC and ACG generally occurred at moderate frequencies. The primary interspecific variation was observed in the identity of the third most abundant trinucleotide motif: AGC ranked third in the FMD, cattle, white-tailed deer, and red deer, whereas ACG ranked third in sheep and goat. Overall, the high-frequency motif composition remained relatively stable across these ruminant genomes.

**Figure 3:**
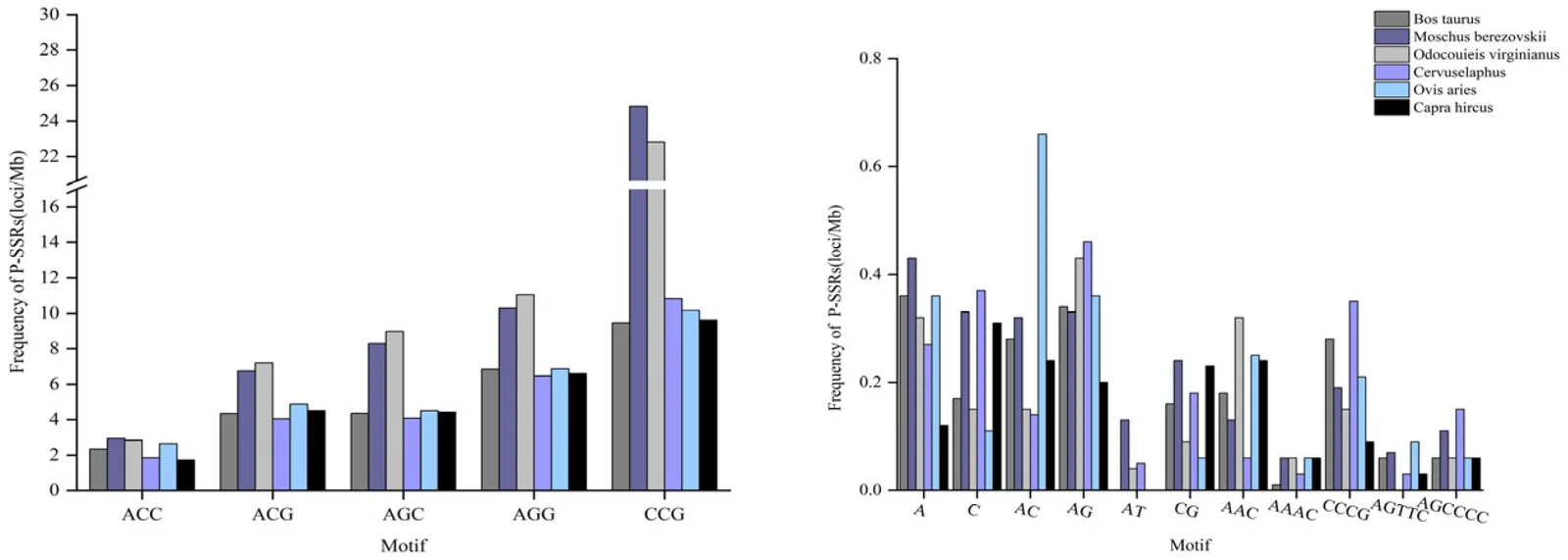
Comparative analysis of SSR abundance across different repeat-copy classes in the protein-coding regions of forest musk deer and its related species.

However, when the comparison was narrowed to motif composition and relative dominance, the FMD displayed more pronounced species-specific features. The most prominent genomic signature was the exceptional enrichment of the CCG motif: the CCG density in the FMD reached 24.82 loci/Mb, the highest among the six species, and was markedly greater than that in cattle (9.46 loci/Mb), red deer (10.82 loci/Mb), sheep (10.15 loci/Mb), and goat (9.62 loci/Mb), though comparable to that in white-tailed deer (22.81 loci/Mb). This divergence indicates that the FMD possesses a strong evolutionary tendency to accumulate GC-rich trinucleotide repeats, potentially associated with local GC content, codon usage bias, or functional constraints acting on specific genes. In addition to CCG, the FMD also exhibited high densities of the other predominant core motifs, AGC and AGG, which were comparable to or higher than those in most other species. Specifically, the AGC density in the FMD (8.97 loci/Mb) was equivalent to that in white-tailed deer (8.97 loci/Mb) and substantially higher than those in cattle (4.35 loci/Mb), sheep (4.50 loci/Mb), red deer (4.08 loci/Mb), and goat (4.42 loci/Mb). Similarly, the AGG density in the FMD (10.29 loci/Mb) was second only to white-tailed deer (11.04 loci/Mb) and exceeded those in cattle (6.84 loci/Mb), sheep (6.86 loci/Mb), red deer (6.46 loci/Mb), and goat (6.60 loci/Mb). These patterns suggest that while the FMD adheres to the conserved ruminant pattern of trinucleotide predominance, its specific motif profile is characterized by a broad enrichment of GC-rich trinucleotides, with CCG showing the most pronounced expansion. Regarding the moderate-frequency motifs ACC and ACG, the FMD was broadly similar to the other species; nevertheless, ACG maintained a comparatively high density in the FMD (6.74 loci/Mb), exceeded only by white-tailed deer (7.20 loci/Mb), indicating that diverse GC-rich trinucleotide repeats are well conserved within its CDSs (Figure 3).

Beyond trinucleotide repeats, the FMD also exhibited distinctive patterns in mononucleotide SSRs: the frequency of A repeats was the highest among all six species (0.43 loci/Mb), while C repeats occurred at a relatively high frequency (0.33 loci/Mb), second only to red deer (0.37 loci/Mb). This indicates that the composition of mononucleotide tracts in the FMD shows a unique enrichment pattern distinct from that observed in other ruminants. Regarding dinucleotide repeats, the density of AC repeats in the FMD (0.32 loci/Mb) was lower than that in sheep (0.66 loci/Mb), but higher than those in cattle (0.28 loci/Mb), goat (0.24 loci/Mb), white-tailed deer (0.15 loci/Mb), and red deer (0.14 loci/Mb). Furthermore, AG repeats were relatively infrequent in the FMD (0.33 loci/Mb) compared with red deer (0.46 loci/Mb), white-tailed deer (0.43 loci/Mb), and sheep (0.36 loci/Mb), whereas AT repeats were present at a low level (0.13 loci/Mb) in the FMD but nearly absent in cattle, sheep, and goat. Notably, CG repeats in the FMD (0.24 loci/Mb) were more abundant than in most other species, a pattern that partially departs from the well-documented suppression of dinucleotide SSRs within coding regions.

For tetranucleotide repeats, motifs such as AAAC, CCCG, and AGTTC were generally rare in the FMD. Although the CCCG motif in the FMD (0.19 loci/Mb) was slightly less frequent than in cattle (0.28 loci/Mb) and red deer (0.35 loci/Mb), its overall abundance remained far below that of the dominant trinucleotide class. Similarly, AGTTC was present at a very low frequency in the FMD (0.07 loci/Mb) and was entirely absent in white-tailed deer. This indicates that higher-order repeats do not constitute a major component of the P-SSR repertoire.

Taken together, although the FMD shares the conserved ruminant framework of trinucleotide predominance in coding regions, it exhibits a distinct motif spectrum characterized by an elevated CCG density, a broad enrichment of GC-rich trinucleotides (including AGC, AGG, and ACG), higher A mononucleotide frequencies, and a relatively abundant CG dinucleotide content, as opposed to the more balanced distribution of multiple trinucleotide motifs observed in white-tailed deer. This GC-rich trinucleotide profile—particularly the marked expansion of CCG—suggests lineage-specific dynamics of microsatellite evolution in the FMD and provides a valuable basis for future marker development and population genetic studies in this species.

### 3.5. Chromosomal Localization Analysis

**Figure 4:**
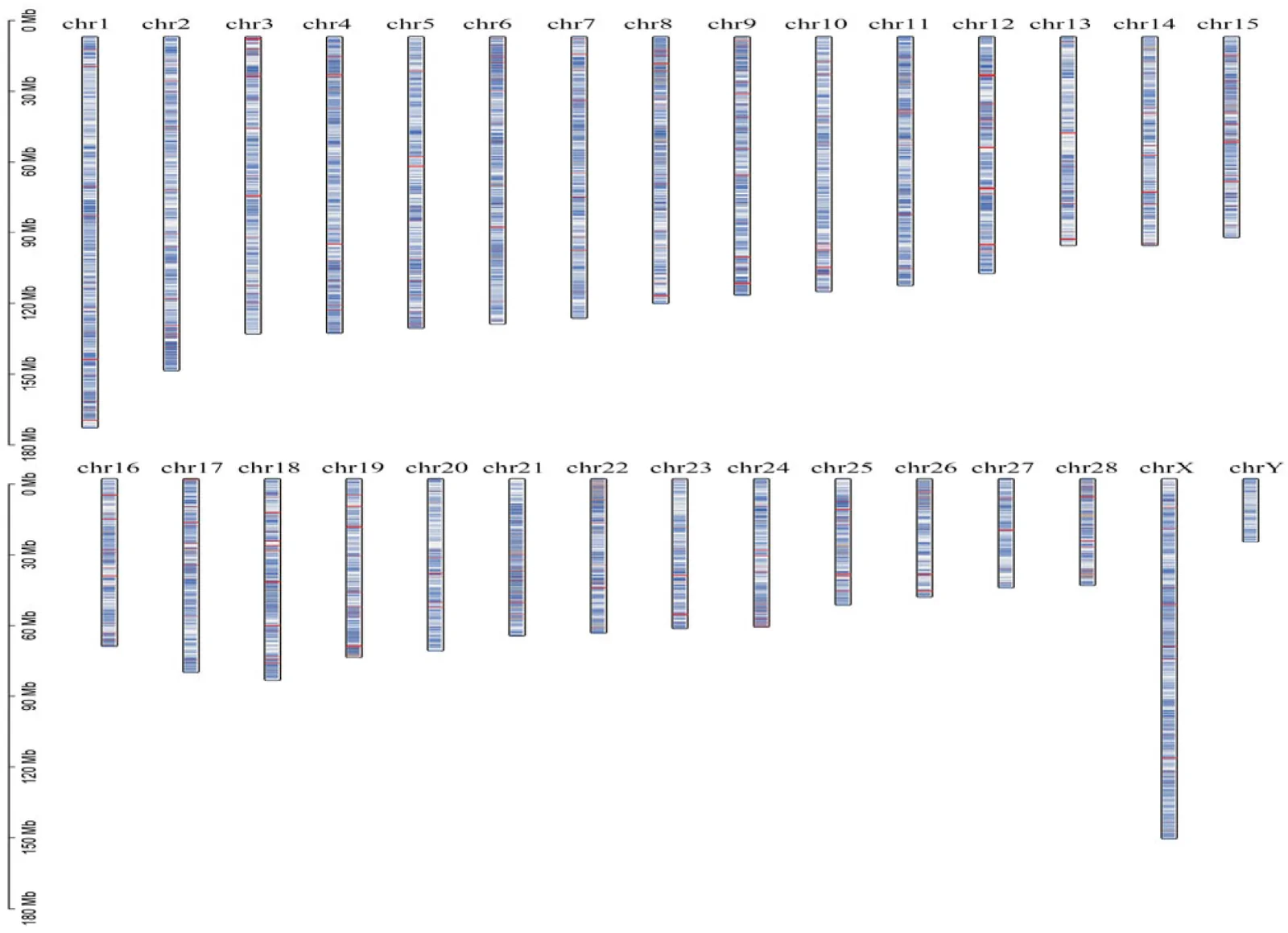
Chromosomal localization of CDS sequences containing SSRs in *M. berezovskii.* Note: blue tracks denote the genome-wide distribution of annotated genes on each chromosome, while red tracks indicate the chromosomal density of genes harboring microsatellites within their CDS regions.

Analysis of the FMD CDSs identified a total of 1766 SSR-containing genes. The physical mapping of these genes across the chromosomes is illustrated in Figure 4, in which blue bands denote the overall gene distribution and red bands highlight the density of SSR-containing CDSs. Notably, these SSR-containing genes were highly enriched on Chr Y, Chr 3, Chr 8, Chr 15, and Chr 28, with the Y chromosome harboring the highest number (47 genes). At the sub-chromosomal level, the majority of SSR-containing genes were localized to the terminal or subtelomeric regions, whereas fewer were found in the interstitial regions. Distinct gene clustering was evident on specific chromosomes, such as Chr 12 and Chr 18, a phenomenon potentially driven by gene duplication events or localized sequence amplification. Furthermore, the detection of SSR-containing genes on the X chromosome confirms their presence on the sex chromosomes in addition to the autosomes, albeit at a lower density. Overall, chromosomal mapping demonstrated that SSR-containing genes are heterogeneously distributed across the FMD genome.

### 3.6. Synteny Analysis Between the FMD and Related Ruminants

Synteny analysis based on SSR-containing genes within CDSs revealed pronounced chromosomal homology and substantial sequence conservation between the FMD and the five comparator ruminants (cattle, sheep, goat, red deer, and white-tailed deer). Pairwise synteny analyses between the FMD and each of the five species were conducted using TBtools to identify homologous genomic regions and visualize chromosomal collinearity.

The synteny analysis identified syntenic blocks between the FMD and the other species in the following quantities: red deer (1468), white-tailed deer (1594), goat (1424), sheep (1483), and cattle (1422). These blocks collectively encompassed all 2509 SSRs in SSR-containing genes identified in the FMD genome, as well as their orthologous SSR-containing genes in cattle (5615), white-tailed deer (4965), red deer (3005), sheep (5708), and goat (5214).

**Figure 5:**
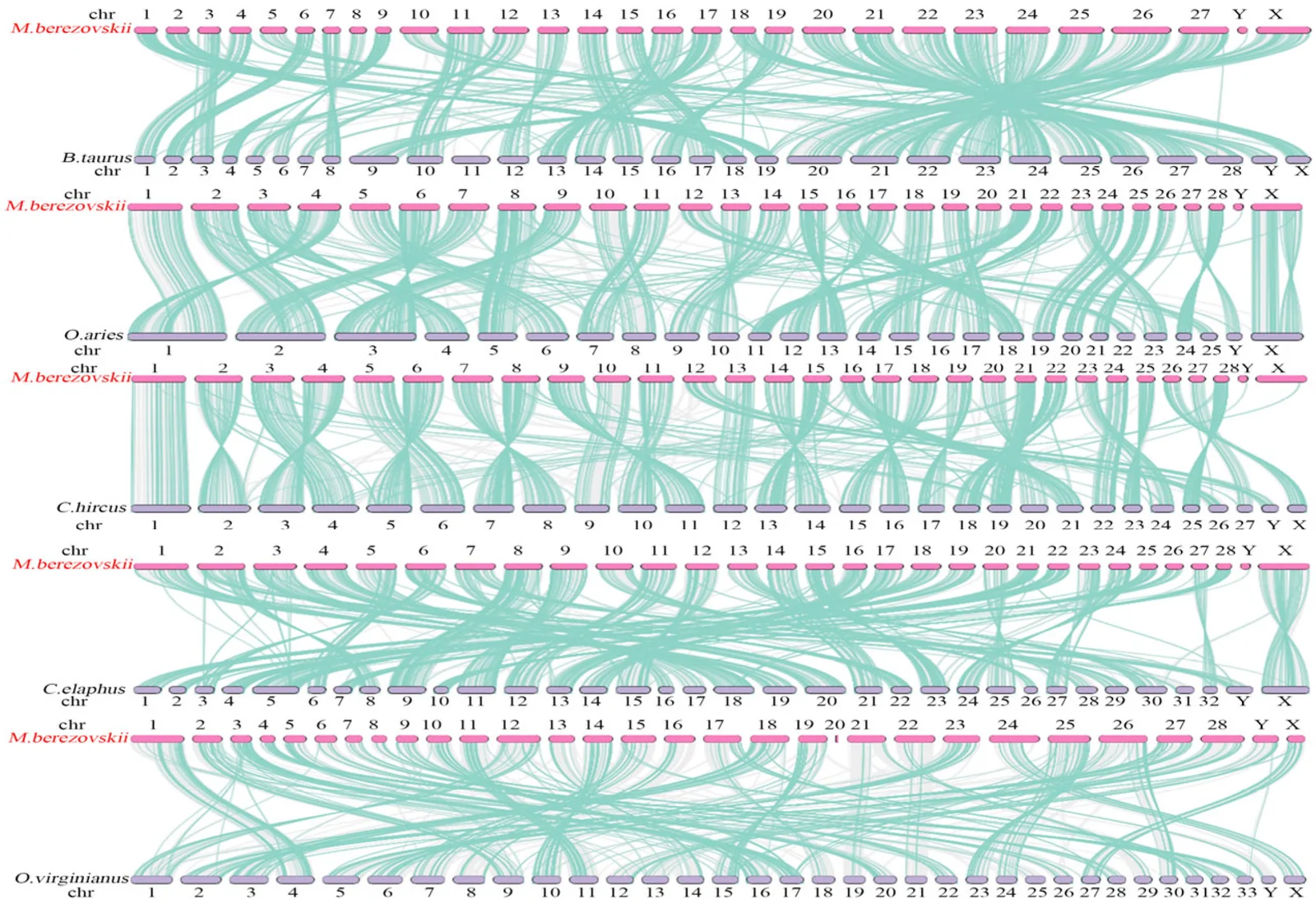
Collinearity analysis of *M. berezovskii* and its closely related species.

In terms of the proportion of syntenic homologs, approximately 48.86% of the FMD SSR-associated genes had collinear counterparts in red deer, followed by 32.10% in Odocoileus virginianus, 27.31% in goat, 25.98% in sheep, and 25.32% in cattle (Figure 5).

Collectively, these results highlight substantial conservation of genome organization among the surveyed ruminants and provide a foundation for further investigation into the evolutionary dynamics of gene functions.

At the chromosomal level, only a small fraction of these syntenic genes were mapped to strictly homologous chromosomes with a 1:1 syntenic correspondence. This pattern indicates that, over evolutionary time, although coding sequences have remained highly conserved at the gene level, the FMD and other artiodactyls have experienced complex chromosomal rearrangements and translocation events. These observations highlight the distinctive architecture of the FMD genome and provide valuable molecular evidence for elucidating its phylogenetic placement.

Further inspection of the spatial distribution of syntenic links in Figure 5 reveals that most homologous gene pairs form interchromosomal connections, visualized as numerous intersecting red linkages. Such connections are particularly prominent between the mid-to-distal regions of FMD chromosomes and multiple chromosomes in the comparator species. A synteny pattern dominated by interchromosomal correspondences suggests frequent genomic structural reshuffling during lineage divergence, potentially involving segmental translocations, inversions, and chromosomal fusions and fissions. In contrast, syntenic relationships retained on homologous chromosomes were relatively limited, reinforcing an evolutionary scenario characterized by highly conserved gene content alongside variable chromosomal localization.

### 3.7. Annotation and Enrichment Analysis

Based on the CDS datasets of the FMD and five related ruminants (white-tailed deer, red deer, sheep, cattle, and goat), we compiled SSR-containing gene sets and performed Gene Ontology (GO) enrichment analysis. The functional profiles were systematically compared across species within the three GO domains: biological process (BP), molecular function (MF), and cellular component (CC).

Overall, the SSR-associated genes exhibited a highly concordant GO enrichment landscape across species, with predominant enrichment in core biological functions related to transcriptional regulation, RNA biosynthesis and processing, DNA binding, and chromatin organization. At the BP level, the enriched terms were consistently dominated by regulation of transcription, DNA-templated; regulation of RNA biosynthetic process; and mRNA processing. At the MF level, enrichment mainly involved DNA binding, transcription factor activity, chromatin binding, and enzyme binding. At the CC level, the SSR-associated genes were primarily enriched in nucleus-related components, including chromosome, nuclear body, and protein complex. Collectively, these results are consistent with the interpretation that SSR-containing genes within coding regions are not randomly distributed with respect to functional categories; rather, they show a statistical concentration in regulatory modules associated with gene expression, which may suggest potential associations with transcriptional control and chromatin-related processes. Notably, this functional pattern appears to be broadly conserved across the examined mammalian species.

Further analysis of the GO terms shared between the FMD and each comparator species revealed marked differences in the extent of overlap. Based on the significantly enriched terms, the largest number of shared GO entries was observed between FMD and red deer (*n* = 51), predominantly involving core functional modules associated with transcriptional regulation, RNA biosynthesis, mRNA processing, DNA binding, and chromatin regulation. The second highest overlap occurred between FMD and white-tailed deer (*n* = 44); in addition to fundamental transcription-related terms, these shared entries also included several categories related to protein modification and enzyme binding.

**Figure 6:**
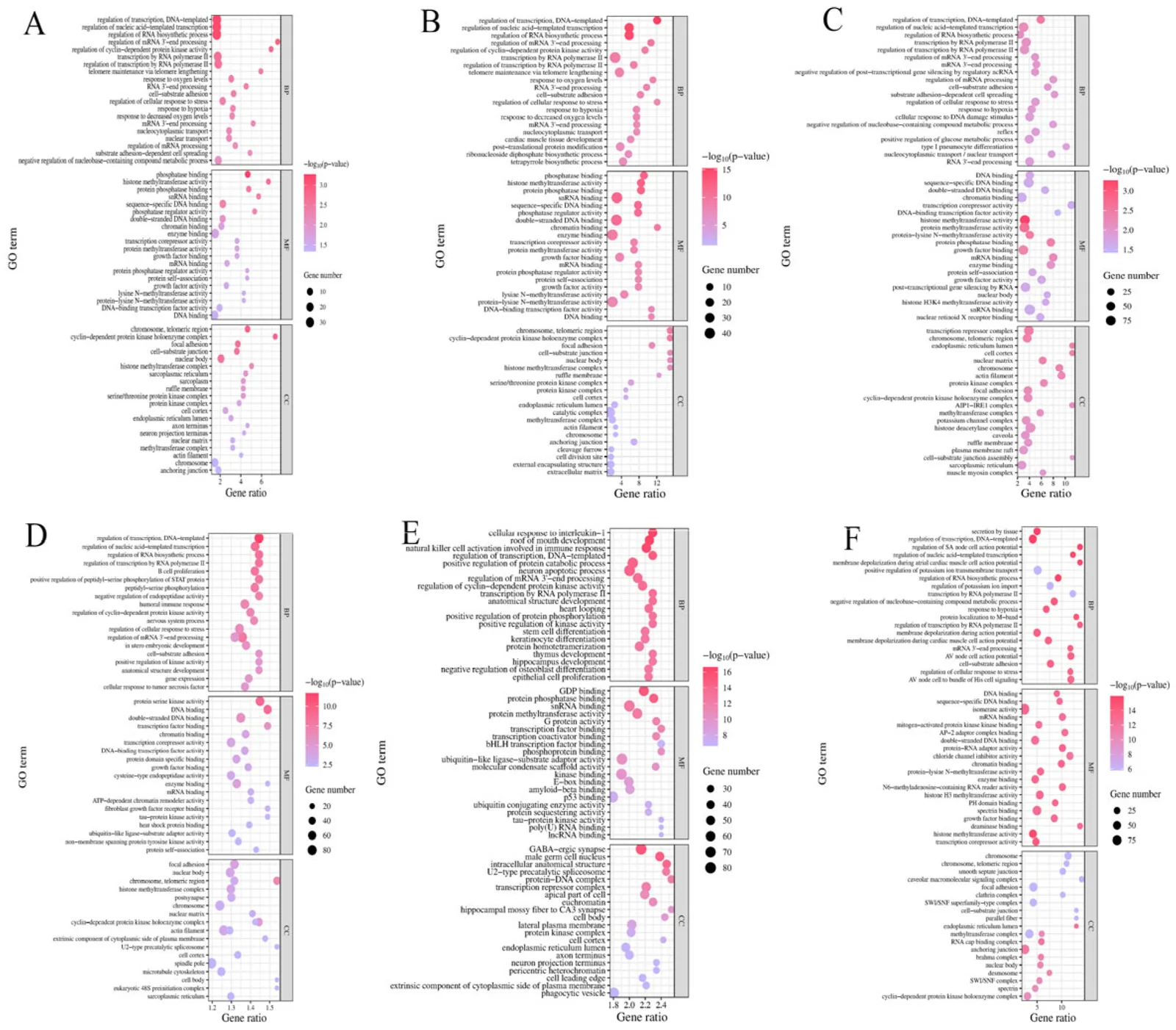
Gene Ontology enrichment analysis of *M. berezovskii* and its closely related species, (**A**) = *M. berezovskii*, (**B**) = *Ce. elaphus*, (**C**) = *Od. virginianus*, (**D**) = *Ov. aries*, (**E**) = *Ca. hircus*, (**F**) = *B. taurus.*

The numbers of shared terms between the FMD and sheep or goat were intermediate (*n* = 22 and *n* = 24, respectively). Although these comparisons retained essential functions such as DNA binding and transcription factor activity, they exhibited more evident divergence in GO terms associated with immune functions and neural regulation. In contrast, the FMD shared more enriched terms with cattle (*n* = 29) than with sheep or goat. This higher overlap is likely attributable to the highly curated and comprehensive annotation of the cattle genome, which yields a larger set of detectable enriched terms, thereby capturing more of the baseline regulatory processes shared with the FMD. The terms shared between the FMD and cattle were concentrated in fundamental transcriptional regulation and RNA processing, whereas greater differences were observed in categories related to ion transport and cardiac muscle function. Overall, the extent of GO-term overlap followed the order: red deer > white-tailed deer > cattle > goat ≈ sheep (Figure 6). These results suggest that the FMD is functionally most similar to cervid species and most divergent from goat and sheep, broadly consistent with their phylogenetic relationships.

Against this generally conserved background, the GO enrichment profile of the FMD exhibited distinct species-specific features. First, within the biological process (BP) domain, the FMD showed significant enrichment for hypoxia- and stress-related terms, including response to hypoxia, response to oxygen levels, and regulation of cellular response to stress, offering preliminary clues that SSR-associated genes may be linked to environmental adaptation in this species. Second, with respect to post-transcriptional regulation, the FMD displayed pronounced enrichment in multiple mRNA/RNA processing terms, such as mRNA 3′-end processing, RNA 3′-end processing, and regulation of mRNA processing, which may be consistent with a more elaborate regulatory architecture involving RNA splicing, modification, and maturation. Moreover, within the cellular component (CC) domain, significant enrichment was observed for telomere-related categories, including chromosomal and telomeric regions as well as telomere maintenance. Together with the enrichment of molecular function (MF) terms such as histone methyltransferase activity and protein phosphatase binding, these findings are compatible with the interpretation that SSR-containing genes in the FMD are associated not only with transcriptional control but also potentially with chromosomal stability and epigenetic regulation.

Cross-species comparisons further revealed distinct functional biases among the ruminants. The white-tailed deer showed stronger enrichment in terms associated with DNA damage response and cellular stress, consistent with a potential emphasis on mechanisms safeguarding genomic integrity. The red deer exhibited more pronounced enrichment in protein modification and metabolism-related categories, consistent with enhanced post-translational regulatory activity. Sheep displayed a relatively high proportion of immune-related GO terms, suggesting potential associations between SSR-containing genes and immune response regulation. Cattle, in contrast, were characterized by enrichment in functions linked to ion transport and cardiac electrophysiology, including potassium ion transmembrane transport and the regulation of cardiomyocyte action potentials, highlighting distinctive physiological regulatory patterns. The goat showed enrichment in nervous system and synapse-related pathways, accompanied by immune-regulatory functions, suggesting potential coordinated modulation between neural and immune processes. Compared with these species-specific tendencies, the FMD did not exhibit an apparent bias toward any single physiological system. Instead, its SSR-containing genes were primarily concentrated in gene expression regulation and environmental response pathways, with particularly strong signals related to hypoxia adaptation and RNA processing regulation. Such interspecific differences in functional enrichment may reflect adaptive divergence potentially shaped by contrasting ecological niches and evolutionary pressures.

Overall, SSRs in CDS-containing genes appear to share a conserved functional framework across species, predominantly centered on transcriptional regulation and chromatin organization. Superimposed on this shared network, each species displays a distinct functional signature potentially driven by the differential enrichment of specific GO categories. While retaining these conserved core pathways, the FMD exhibits markedly enhanced enrichment in hypoxia response, RNA processing, and epigenetic regulation, which may provide preliminary insights into potential molecular adaptations associated with its specialized ecological niche. It should be emphasized that these GO-based associations represent correlational hypotheses rather than evidence of causal roles for SSRs in driving adaptive evolution.

### 3.8. KEGG Pathway Enrichment Analysis

**Figure 7:**
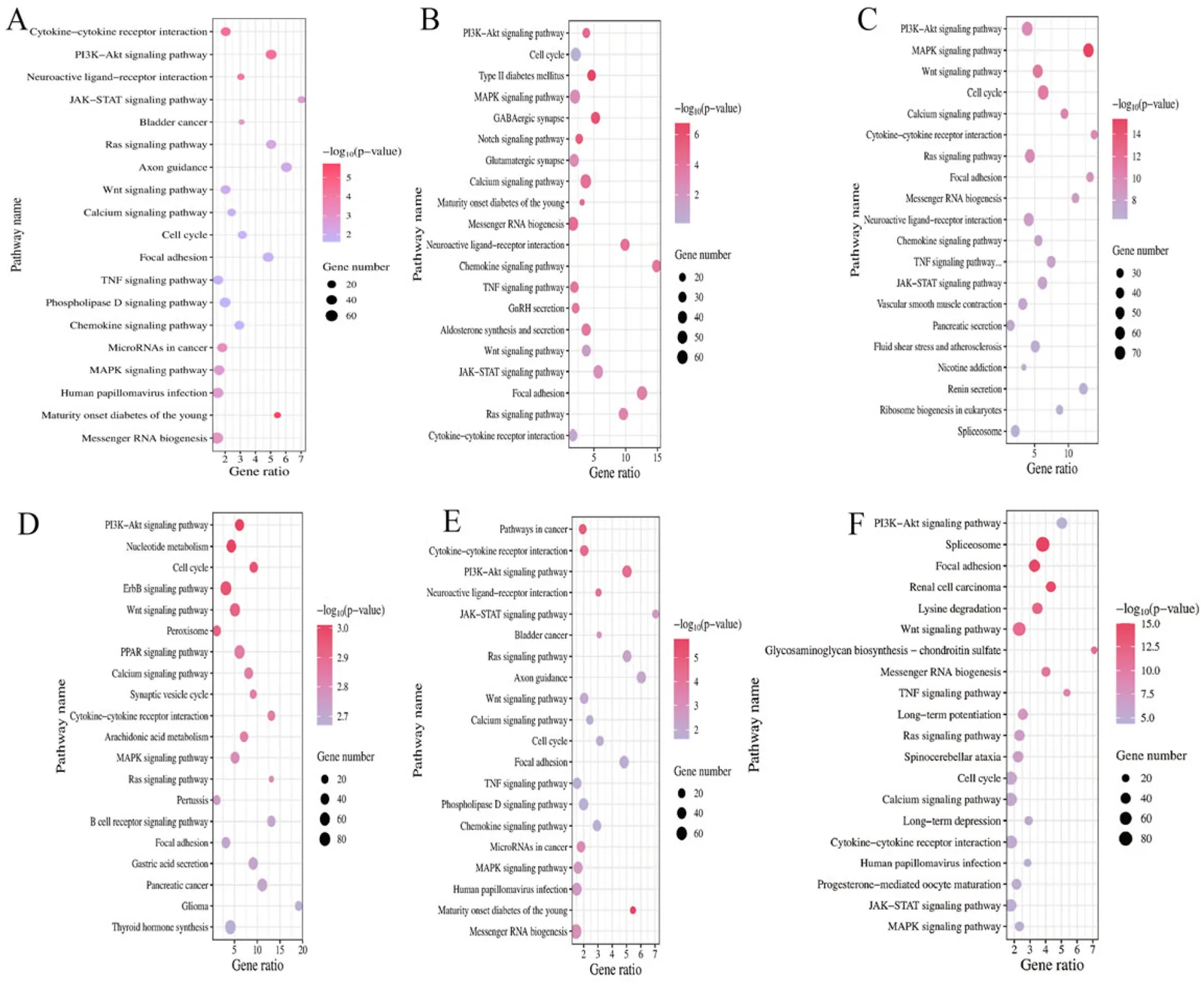
KEGG enrichment analysis of CDS-SSR related genes in *M. berezovskii* and closely related ruminant species. (**A**) = *M. berezovskii*; (**B**) = *Ce. elaphus*; (**C**) = *Od. virginianus*; (**D**) = *Ov. aries*; (**E**) = *Ca. hircus*; (**F**) = *B.taurus*.

KEGG pathway enrichment analysis of SSRs in CDS-containing genes in the FMD revealed that the significantly enriched pathways were predominantly concentrated in canonical signaling axes, including the PI3K-Akt, MAPK, Ras, Wnt, and JAK-STAT signaling pathways. These were accompanied by enrichment in the cell cycle, calcium signaling, focal adhesion, and cytokine–chemokine-related pathways. Collectively, these findings are consistent with a potential concentration of SSR-containing genes in pathways associated with growth and development, immune responses, and inflammation (Figure 7).

The FMD and red deer shared 14 enriched pathways, including cytokine–cytokine receptor interaction, chemokine signaling pathway, TNF signaling pathway, JAK-STAT signaling pathway, PI3K-Akt signaling pathway, MAPK signaling pathway, Ras signaling pathway, Wnt signaling pathway, cell cycle, calcium signaling pathway, focal adhesion, neuroactive ligand–receptor interaction, mRNA biogenesis, and maturity-onset diabetes of the young, consistent with highly similar enrichment patterns in cytokine networks, cell proliferation, and signal transduction. In addition, red deer exhibited enrichment in the Notch signaling pathway, GABAergic synapse, glutamatergic synapse, GnRH secretion, aldosterone synthesis and secretion, and type II diabetes mellitus, consistent with a potential emphasis on neuroendocrine and metabolic regulation.

The FMD and white-tailed deer shared 13 enriched pathways, likewise covering the PI3K-Akt, MAPK, Ras, and Wnt signaling pathways; the JAK-STAT signaling pathway; the cell cycle; calcium signaling pathway; and focal adhesion, as well as immune and inflammatory pathways such as cytokine–cytokine receptor interaction, chemokine signaling pathway, and TNF signaling pathway, while also retaining categories related to signal transduction and post-transcriptional regulation (e.g., neuroactive ligand–receptor interaction and mRNA biogenesis). Meanwhile, white-tailed deer showed additional enrichment in vascular smooth muscle contraction, pancreatic secretion, fluid shear stress and atherosclerosis, and renin secretion, as well as ribosome biogenesis in eukaryotes and spliceosome, consistent with a more prominent pathway spectrum associated with vascular physiology and protein synthesis and splicing.

Notably, among the comparisons with cervids, the FMD uniquely displayed enrichment in the phospholipase D signaling pathway, microRNAs in cancer, and human papillomavirus infection, which were not among the leading enriched pathways in either red deer or white-tailed deer. This pattern is consistent with the possibility that SSR-associated genes in the FMD may be linked to membrane lipid-mediated signaling, as well as to post-transcriptional and cancer-related regulatory networks.

The sheep and the FMD shared eight enriched pathways, which largely overlapped in core growth- and proliferation-related signaling modules, including the PI3K-Akt, MAPK, Ras, and Wnt signaling pathways; the cell cycle; calcium signaling pathway; focal adhesion; and cytokine–cytokine receptor interaction. This overlap suggests that their common enrichment patterns remain centered on cell-cycle control and key growth signaling axes. In contrast, the sheep enrichment profile additionally featured multiple metabolism-oriented modules, such as nucleotide metabolism, peroxisome, PPAR signaling pathway, and arachidonic acid metabolism, and also included pathways related to immunity and endocrine and digestive regulation (e.g., B cell receptor signaling pathway, pertussis, thyroid hormone synthesis, and gastric acid secretion). Collectively, these results are compatible with the interpretation that SSR-associated genes in the sheep may be statistically associated with energy homeostasis and humoral immune processes. This shift may potentially contribute to the relatively reduced overlap with the FMD in more specific immune–inflammatory pathways (e.g., TNF and chemokine-related pathways, and JAK-STAT signaling), as well as in post-transcriptional processes such as mRNA biogenesis. The goat exhibited the greatest overlap with the FMD, sharing 19 enriched pathways. These essentially encompassed the full set of FMD-enriched categories, including the cytokine-, chemokine-, and TNF-related pathways; the PI3K-Akt, MAPK, Ras, and Wnt signaling axes; JAK-STAT signaling pathway; the cell cycle; calcium signaling pathway; focal adhesion; axon guidance; phospholipase D signaling pathway; microRNAs in cancer; human papillomavirus infection; bladder cancer; mRNA biogenesis; and maturity-onset diabetes of the young, with pathways in cancer additionally enriched as a higher-level integrative category. This highly concordant pathway spectrum is compatible with the interpretation that the functional enrichment direction of SSR-associated genes is highly similar between the FMD and the goat, particularly with respect to the coordinated enrichment of immune and inflammatory cytokine networks and multiple growth-related signaling cascades. Such concordance is compatible with the interpretation that SSR loci in both species may preferentially occur within conserved, pleiotropic regulatory gene sets.

The cattle and the FMD shared 12 enriched pathways, with the overlapping categories mainly concentrated in the PI3K-Akt, MAPK, Ras, Wnt, JAK-STAT, and TNF signaling pathways; cytokine–cytokine receptor interaction; the cell cycle; calcium signaling pathway; focal adhesion; human papillomavirus infection; and mRNA biogenesis. This pattern suggests that the cattle and the FMD likewise retain a relatively stable ruminant core framework centered on signal transduction, immune and inflammatory regulation, and cell proliferation and adhesion. However, the cattle-specific enrichment profile showed a stronger bias toward pathways related to structural maintenance, post-transcriptional regulation, and reproductive and neural functions, including spliceosome, glycosaminoglycan biosynthesis–chondroitin sulfate, lysine degradation, renal cell carcinoma, long-term potentiation, long-term depression, spinocerebellar ataxia, and progesterone-mediated oocyte maturation. These results are compatible with the interpretation that SSR-associated genes in the cattle display more prominent enrichment signals in RNA splicing, extracellular matrix and tissue-structure maintenance, as well as in neuroreproductive regulatory processes.

Taken together, the KEGG profiles of all six species show that pathways such as the PI3K-Akt, MAPK, Ras, and Wnt signaling pathways; the cell cycle; calcium signaling pathway; and focal adhesion recurred across multiple species, constituting a conserved cross-species backbone network. Against this shared framework, each species developed its own enrichment emphasis through the differential expansion of immune and inflammatory pathways (e.g., cytokine-, chemokine-, TNF-, and JAK-STAT-related pathways), together with variation in post-transcriptional, metabolic, and endocrine and neural pathways. These findings are compatible with the interpretation that SSRs in CDS-associated genes not only reflect the highly conserved key biological processes shared among ruminants, but may also be associated with species-specific differentiation in immune adaptation, metabolic strategy, and physiological function in the context of particular gene sets embedded within these signaling pathways and regulatory networks.

### 3.9. Primer Design and Screening

After multiple rounds of stringent parameter optimization, 20 primer pairs that met the design criteria were generated. Following specificity screening, five highly specific primer pairs were selected for subsequent amplification and validation (Table 4).

These five primer pairs were then used to amplify genomic DNA from 24 FMD individuals by PCR. After passing quality control, the PCR products were submitted to a commercial genotyping service for capillary electrophoresis, which enabled accurate fragment-size determination and allele calling. This step served as the final validation of primer performance and provided the essential dataset for a robust evaluation of genetic polymorphism in the FMD population. Based on the initial genotyping of the 24 individuals, allele distributions at each locus were directly assessed, confirming that the selected primer pairs exhibited satisfactory polymorphism and stable amplification in practical applications.

### 3.10. PCR Amplification and Electrophoretic Validation

**Figure 8:**
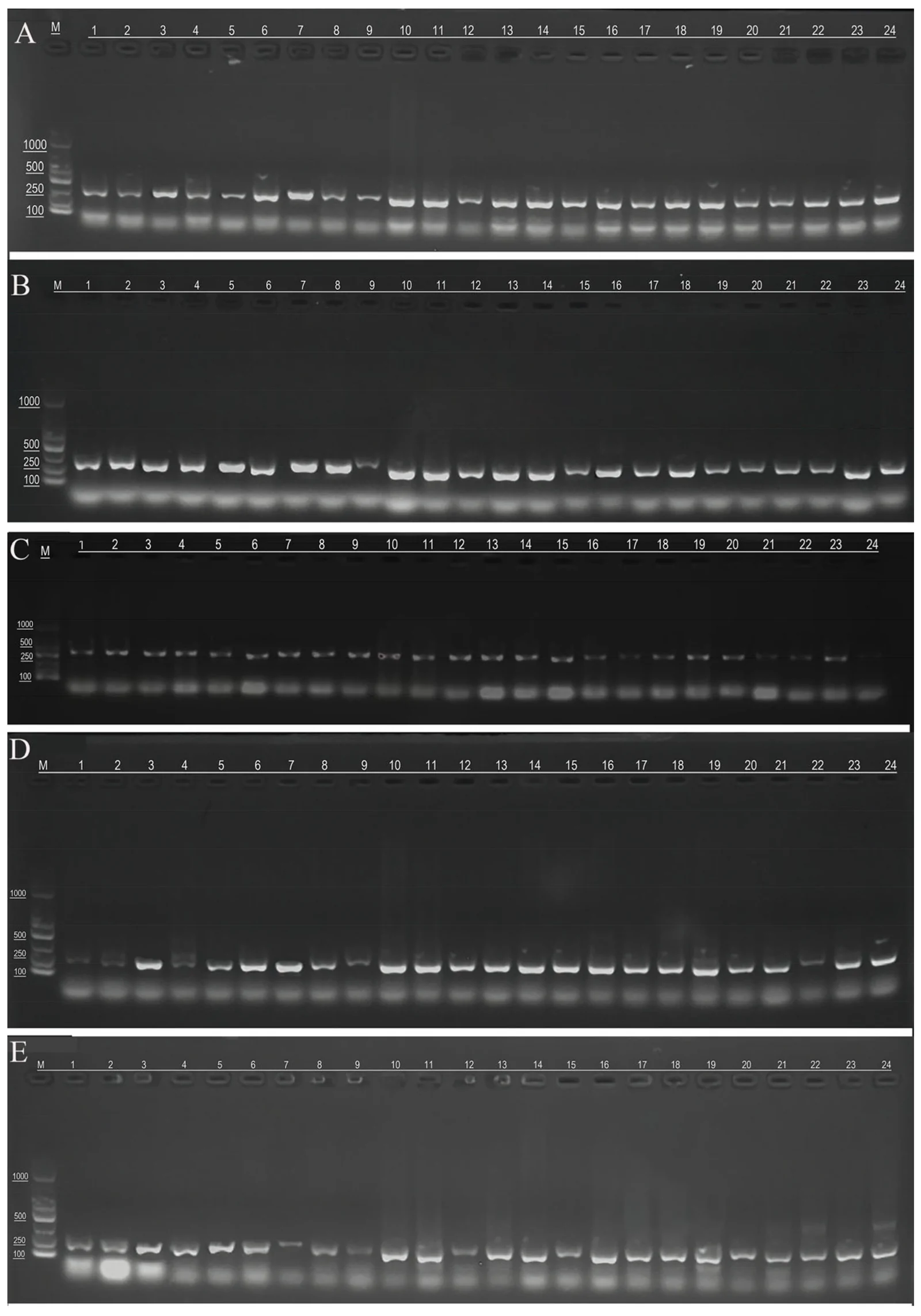
Agarose gel electrophoresis of PCR products amplified by five CDS-derived SSR markers in *M. berezovskii.* Note: (**A**): Mberchr1T0302; (**B**): Mberchr25T0046; (**C**): Mberchr2T0168; (**D**): Mberchr22T0827; (**E**): Mberchr2T0740.

To evaluate amplification efficiency and primer specificity, PCR validation was performed for the five selected SSR loci: Mberchr25T0046, Mberchr2T0168, Mberchr1T0302, Mberchr2T0740, and Mberchr22T0827. Electrophoretic analysis demonstrated that all five loci yielded clear and distinct bands across all sampled individuals. The consistent amplification and high reproducibility, with no instances of amplification failure, indicate that the designed primers exhibit robust amplification performance (Figure 8). Furthermore, the amplicons of each locus exhibited high consistency in size and electrophoretic mobility, conforming to the expected design ranges without significant deviations.

No conspicuous non-specific amplification or severe smearing was observed across the loci. The minor variations in band intensity detected in a few samples were attributed to slight differences in template DNA concentration or reaction components, and did not interfere with the overall interpretation of the results. In addition, the amplicons were characterized by sharp bands and low background noise, confirming a well-optimized PCR system and high primer specificity. The absence of visible primer dimers further indicates an appropriate primer design with minimal non-specific annealing.

In summary, these five SSR loci exhibited robust amplification and high specificity, providing a reliable molecular foundation for subsequent analyses of genetic diversity and population genetic structure in the FMD.

### 3.11. Population Genetic Analysis

To assess the genetic diversity of a captive FMD population, 24 individuals were genotyped at the five newly selected SSR loci. The number of alleles (N*a*) per locus ranged from 17 to 28, with a mean of 22.0, demonstrating substantial allelic diversity across these markers. The observed heterozygosity (H*o*) ranged from 0.23 to 0.61 (mean = 0.40), whereas the expected heterozygosity (H*e*) varied from 0.90 to 0.96 (mean = 0.93). Furthermore, the polymorphism information content (PIC) values ranged from 0.89 to 0.96, with a mean of 0.93. These indices confirm that all selected microsatellites are highly polymorphic, providing strong discriminatory power and high informativeness, and are therefore well suited for population genetic investigations in this species.

Further estimation of the inbreeding coefficient (FIS) revealed values ranging from 0.34 to 0.74, with a mean of 0.57 (Table 5). Positive FIS values were consistently observed across all loci, indicating that the observed heterozygosity was substantially lower than expected and reflecting a pronounced heterozygote deficiency. Such a deficit is common among captive populations and is typically attributable to a limited number of founders, a restricted effective population size, and non-random mating under artificial breeding regimes. Furthermore, cumulative inbreeding over long-term captive propagation has likely contributed to the erosion of genetic heterozygosity. Nevertheless, the newly characterized SSR loci retained high allelic richness and polymorphism information content (PIC), underscoring their robust utility for genetic diversity assessment, population structure analysis, and individual identification in the FMD. Consequently, future conservation and breeding programs should incorporate stringent genetic monitoring and optimized mating designs to preserve the existing genetic diversity and mitigate potential inbreeding risks [37].

## 4. Discussion

This study comparatively analyzed CDS-derived SSRs in the FMD and closely related ruminants. While the predominance of tri- and hexanucleotide repeats across all six species aligns with the triplet genetic code and purifying selection against frameshift mutations [3,6,32,38,39,40,41,42,43], the cross-species comparison reveals nuanced differences in how these constraints manifest across lineages. Notably, the FMD combined a conserved ruminant framework with distinct features—specifically, the highest pentanucleotide proportion and marked CCG enrichment—suggesting that mutational and selective pressures may have operated differently on its coding microsatellite repertoire [8,44,45]. Synteny analysis indicates that SSR-containing genes are embedded within chromosomal segments that remain largely conserved at the sequence level across ruminants, despite extensive structural rearrangement [46,47,48,49]. The closer homology between the FMD and cervids is consistent with phylogenetic expectations, but the prevalence of interchromosomal syntenic links implies that these functionally important genes have been dynamically repositioned through fission, fusion, and translocation [50,51]. This dichotomy—high gene-level conservation coupled with fluid chromosomal architecture—suggests that CDS-SSRs reside in genomic neighborhoods under strong functional constraint, a pattern that facilitates cross-species marker transfer while preserving lineage-specific variation.

From a conservation and comparative genomics perspective, the deep conservation of CDS-SSR loci is practically significant: well-characterized markers from model ruminants (e.g., cattle, goat) can inform primer design and genetic monitoring in endangered relatives such as the FMD. Conversely, the FMD-specific motif profile and functional associations (hypoxia response, epigenetic regulation, and RNA processing) provide preliminary clues to molecular adaptations in this montane forest specialist. Collectively, these findings offer a dual perspective—conserved microsatellite architectures that enable comparative genomic inference, alongside species-specific signatures that may reflect ecological adaptation—thereby establishing both a reference framework for ruminant evolutionary genomics and practical molecular tools for the genetic management of captive FMD populations.

Functionally, KEGG enrichment analysis showed that CDS-associated SSRs in all six species were predominantly enriched in canonical signaling pathways, including PI3K-Akt, MAPK, Ras, and Wnt, a pattern consistent with the possibility that these core regulatory networks have been highly conserved throughout ruminant evolution [21,52], although the enrichment itself does not establish a causal role of SSRs in pathway function. These pathways are known to participate extensively in cell proliferation, immune regulation, tissue development, and metabolic homeostasis, and may contribute to the molecular machinery underlying growth and environmental adaptation [53,54]; for instance, transcriptomic analyses in goat have identified significant enrichment of MAPK, Wnt, and neuroactive ligand–receptor interaction pathways associated with hair follicle development and tissue proliferation [55], supporting the broader relevance of these pathways in ruminant physiology, while comparative genomic analyses have revealed numerous conserved homologous gene clusters and shared KEGG modules among cattle, sheep, and cervids [56], underscoring the conserved genomic architecture of these lineages. Pairwise comparisons revealed that the FMD shared the greatest total number of enriched pathways with goat (19 common pathways), which may reflect broad similarity in immune-inflammatory processes and coordinated regulation across multiple signaling axes; nevertheless, within specific functional categories—particularly immune-factor networks and neuroendocrine-related pathways—the FMD exhibited greater overlap with red deer and white-tailed deer, a pattern consistent with the close phylogenetic affinity among cervids [57]. Species-specific differences were also evident, as cattle and sheep exhibited stronger enrichment in modules related to metabolism, extracellular matrix organization, and endocrine regulation, potentially reflecting divergent selective pressures associated with domestication. These findings are compatible with the interpretation that, although ruminants maintain strong conservation of core signaling pathways, individual species may have developed distinct functional profiles potentially shaped by ecological adaptation, domestication-driven selection, and physiological divergence. Thus, the distribution of SSR-associated genes in ruminants is compatible with an evolutionary scenario characterized by the coexistence of functional conservation and lineage-specific differentiation; however, it should be emphasized that the functional associations identified by GO and KEGG enrichment represent statistical correlations rather than causal relationships, and whether SSRs within these CDS regions actively participate in pathway regulation, alter protein function, or are selectively neutral passengers within functionally important genes remains to be experimentally validated.

Among the reproductive and physiological traits underpinned by these conserved pathways, musk secretion stands out as a unique, male-limited phenotype with profound implications for breeding management. Notably, musk secretion plays critical roles in chemical communication, territorial marking, and mate attraction. Although this study did not identify direct associations between specific CDS-SSR loci and musk yield, SSR-containing genes within the enriched endocrine signaling and cell-proliferation pathways are fundamentally involved in glandular morphogenesis and secretory activity [32]. Collectively, these findings offer preliminary molecular insights into the genetic regulation of musk secretion and further suggest CDS-SSR loci as candidate regulators of musk gland development and function [1]. Moving forward, these markers should be integrated with transcriptomic, proteomic, and phenotypic data—including musk production records, reproductive metrics [58], and behavioral assessments—to facilitate robust identification of genes controlling musk secretion and fertility [59].

Beyond functional insights, the practical utility of CDS-SSR markers for population genetic management requires empirical validation [60,61]. In this study, novel markers were developed from conserved CDS-SSR loci and applied to evaluate the genetic diversity of a captive FMD population. The genotyping results revealed substantial polymorphism across the tested loci, with high polymorphism information content values and heterozygosity levels, indicating robust utility for genetic diversity assessment, marker-assisted breeding, and parentage identification [62]. However, observed heterozygosity was lower-than-expected heterozygosity, a pattern consistent with previous reports in captive FMD populations and indicative of inbreeding and genetic drift [63,64,65]. In light of these findings, continuous genetic monitoring employing highly polymorphic CDS-SSR markers is essential for evaluating population genetic health, optimizing mating strategies, and mitigating inbreeding risks [66,67]. Integrating such genetic assessment with routine health surveillance, breeding records, and behavioral monitoring establishes a multidimensional framework that supports evidence-based management, sustains health resilience, and ensures the long-term welfare and viability of captive stocks [28,68].

## 5. Conclusions

This study presents a comprehensive comparative genomic analysis of coding-sequence microsatellites (CDS-SSRs) in the FMD and five closely related ruminant species. Our results indicate that CDS-SSRs follow broadly conserved evolutionary patterns across ruminants, with trinucleotide repeats predominating in coding regions—a distribution consistent with stringent purifying selection against frameshift mutations. In contrast, the FMD exhibits distinct lineage-specific features, including a markedly elevated relative density of CDS-SSRs and significant enrichment of CCG repeat motifs, consistent with unique microsatellite evolutionary trajectories and potential genomic adaptations.

Functional annotation indicated that SSR-containing genes were predominantly associated with transcriptional regulation, RNA processing, chromatin organization, and multiple conserved signaling pathways, including PI3K-Akt, MAPK, Ras, Wnt, and JAK-STAT. These findings are compatible with the interpretation that CDS-SSRs are located within genes participating in regulatory networks governing growth, development, stress responses, and immune functions; however, these positional associations do not establish that the SSRs themselves are functional elements, and their potential roles as neutral passengers, regulatory modulators, or structural variants remain to be experimentally distinguished. At the chromosomal level, synteny analysis further supported substantial conservation of SSR-associated genes between the FMD and related ruminants, alongside lineage-specific genomic features.

To translate these genomic findings into conservation practice, we developed and validated five highly polymorphic CDS-SSR markers in a captive FMD population. While genotyping with these markers captured substantial genetic variation, it also revealed marked heterozygote deficiency and elevated inbreeding coefficients—clear indicators of genetic erosion risk under prolonged closed breeding. These markers thus provide immediately applicable molecular tools for routine genetic monitoring, pedigree reconstruction, and evidence-based optimization of breeding strategies.

Collectively, this study provides insights into the evolutionary constraints and positional distribution of coding-region microsatellites across ruminant genomes, while delivering actionable molecular resources for the conservation and scientifically informed management of captive FMD populations.

## Figures and Tables

**Table 1:** Primer Design for CDS Regions of *M. berezovskii*.

Gene Name	Primer	TM (°C)	Core Repeat	Fragment Length (bp)
Mberchr1T0302	F: TGTCGTCGGTCGGCTCCATTC	55.0 °C	(CGG)_n_	233
	R: CTTGGCGTCGGAGGTGAGGC			
Mberchr22T0827	F: CTGCCGCCTCCAGTAGCCA	59.5 °C	(ACC)_n_	244
	R: CGAGAACGGGTGCTGGAAGTA			
Mberchr25T0046	F: ATGGCATCCCAGGAGTGT	59.0 °C	(GAG)_n_	270
	R: TTCAGTGGAGATCGGTAGTG			
Mberchr2T0740	F: AGCCAAACCAAGTCACTATCA	55.0 °C	(GGT)_n_	243
	R: TCAAGGTCACTGCGGATG			
Mberchr2T0168	F: ATGCACTCGACCCAGATCAC	56.5 °C	(GGC)_n_	299
	R: AGGCTCGAGGTCTTCCTTCT			
Mberchr1T0298	F: TGAAGATTACGCCTGTGG	50.0 °C	(TTC)_n_	282
	R: GCAATGGGATTTGACACC			
Mberchr4T0944	F: GTGCCCACATTTGTTTCT	51.7 °C	(CAG)_n_	327
	R: ATCTGCTCGGACCATCTT			
MberchrXT0127	F: GTTAGTGCCTGTGACCCG	53.3 °C	(GAG)_n_	206
	R: GAAATGCGATGTTCTCCC			
Mberchr24T0194	F: AGACCGCCTACTGCGACAT	57.0 °C	(GAG)_n_	291
	R: TGAATTGGCTCTTCCTTTGG			
Mberchr1T0296	F: AGGAACTCCAGGAAGAAG	49.3 °C	(GAG)_n_	246
	R: CAAGAAGACGGAAATCAA			
Mberchr4T0395	F: TTACTTTCCAGAACCCTTACT	52.4 °C	(GCA)_n_	252
	R: CCTGGGAATGATTGTAGC			
Mberchr4T0495	F: AGTTGGGCAAGTTCCTCC	52.0 °C	(CAA)_n_	337
	R: GGGTATCCCTGGTATTCG			

**Table 2:** Overview of the CDS of *M. berezovskii* and its closely related species.

Parameters	*M. berezovskii*	*Od. virginianus*	*Ce. elaphus*	*B. taurus*	*Ov. aries*	*Ca. hircus*
Total number of genes of CDS	24,375	20,293	23,212	21,840	21,550	20,728
GC content (%)	53.09	54.08	54.07	53.76	53.82	53.96
Number of SSRs	2325	2248	2477	2295	2547	2176
Total length of SSRs (bp)	47,903	40,952	44,173	40,797	46,395	39,670
CDS SSR content (%)	0.13	0.12	0.12	0.12	0.13	0.12
Relative abundance (Loci/Mb)	62.61	65.49	66.17	63.32	70.64	62.44
Relative density (bp/Mb)	1289.93	1193.01	1180.03	1125.56	1286.82	1138.37

**Table 3:** Abundance of various microsatellite types in CDS regions of six ruminant species (loci/Mb).

Type	*M. berezovskii*	*Ce. elaphus*	*Od. virginianus*	*B. taurus*	*Ov. aries*	*Ca. hircus*
CD-SSRs	0.38	0.19	0.41	0.36	0.44	0.34
CX-SSRs	0.00	0.03	0.03	0.00	0.03	0.00
ICD-SSRs	1.00	0.88	0.82	0.72	0.94	0.72
ICX-SSRs	0.24	0.13	0.23	0.11	0.14	0.14
IP-SSRs	0.81	0.56	0.73	0.88	0.97	0.86
P-SSRs	62.61	66.17	65.49	63.32	70.64	62.44
Total	65.04	67.96	67.71	65.39	73.16	64.50

Note: Compound, CD; Interrupted Compound, ICD; Complex, CX; Interrupted Complex, ICX; Perfect, P; Interrupted Perfect, IP.

**Table 4:** Characteristics of 5 polymorphic SSRs in CDS markers developed for *M. berezovskii.*

Gene Name	Primer	TM (°C)	Fluorescent Labeling	Core Repeat	Fragment Length
Mberchr1T0302	F: TGTCGTCGGTCGGCTCCATTC	55.0 °C	FAM	(CGG)_n_	233
	R: CTTGGCGTCGGAGGTGAGGC				
Mberchr22T0827	F: CTGCCGCCTCCAGTAGCCA	59.5 °C	TAM	(ACC)_n_	244
	R: CGAGAACGGGTGCTGGAAGTA				
Mberchr25T0046	F: ATGGCATCCCAGGAGTGT	59.0 °C	FAM	(GAG)_n_	270
	R: TTCAGTGGAGATCGGTAGTG				
Mberchr2T0740	F: AGCCAAACCAAGTCACTATCA	55.0 °C	HEX	(GGT)_n_	243
	R: TCAAGGTCACTGCGGATG				
Mberchr2T0168	F: ATGCACTCGACCCAGATCAC	56.5 °C	TAM	(GGC)_n_	299
	R: AGGCTCGAGGTCTTCCTTCT				

**Table 5:** Genetic diversity indices of five CDS-derived SSR markers in *M. berezovskii.*

Locus ID	Na	Ho	He	PIC	Fis
Mberchr1T0043	22	0.35	0.93	0.92	0.62
Mberchr25T0046	22	0.61	0.93	0.92	0.34
Mberchr2T0740	21	0.47	0.95	0.94	0.51
Mberchr22T0827	17	0.23	0.90	0.89	0.74
Mberchr2T0168	28	0.33	0.96	0.96	0.66
Mean	22.0	0.40	0.93	0.93	0.57

## Data Availability

The original contributions presented in this study are included in the article/Supplementary Material. Further inquiries can be directed to the corresponding author(s).

## Supplementary Materials

The following supporting information can be downloaded at: https://www.mdpi.com/article/10.3390/vetsci13080808/s1, Table S1: Summary of reference genome assemblies of ruminant species used in this study.
