# Supplementary material for: Comparative Genomic Analysis of Coding Sequence-Derived Microsatellites Reveals Evolutionary Conservation and Genetic Diversity in Forest Musk Deer (Moschus berezovskii) and Related Ruminants
Source: Vet Sci. 2026 Aug 15;13(8):808. doi: 10.3390/vetsci13080808 (PMC13517226; doi:10.3390/vetsci13080808)
Supplement: Supplementary file 1 [file vetsci-13-00808-s001.zip › vetsci-4479269Supplementary File .pdf]

Table S1. Summary of reference genome assemblies of ruminant species used in this study.

	Assembly Versions	Accession Numbers	Annotation Releases	Assembly Level	Download Dates
Moschus berezovskii	MUSKDBV1.0	GCF_022376915.1	RS-2023-04	Chromosome	2025.09.15
Bos taurus	ARS-UCD2.0	GCF_002263795.3	RS-2024-12	Chromosome	2025.09.15
Ovis aries	ARS-UI_Ramb_v3.0	GCF_016772045.2	RS-2023-10	Chromosome	2025.09.15
Capra hircus	ARS1.2	GCF_001704415.2	RS-2016-08	Chromosome	2025.09.16
Cervus elaphus	mCerEla1.1	GCF_910594005.1	RS-2021-07	Chromosome	2025.09.16
Odocoileus virginianus	Ovbor_1.2	GCF_023699985.2	RS-2024-12	Chromosome	2025.09.16
